# The 1922 Zionist launch of sustainable malaria control and an examination of the education which enabled that control

**DOI:** 10.5281/zenodo.15389413

**Published:** 2025-05-12

**Authors:** Anton Alexander, Bart G.J. Knols

**Affiliations:** 1 BC Business Centrum, Elscot House, Arcadia Avenue, London N3 2JU, United Kingdom.; 2 Editor, MalariaWorld Journal.

## Abstract

This paper examines why there was the necessity for prioritising sustainable malaria control in Palestine in 1922. It then follows by reviewing the method employed by the Zionists to achieve that goal. It also examines the difficulties encountered in organising education for the inhabitants and further examines evidence of the effectiveness of the education.

## Background

The Panama Canal was considered the construction miracle of the beginning of the 20th century. At the time, it also was a demonstration of malaria and yellow fever management based on rigorous mosquito abatement enforced through military discipline, and it will also be a useful starting point for the reader of this paper to appreciate the approach then employed of the first major attempt at malaria control.

For many years, consideration had been given to the construction of a canal to connect the Caribbean Sea and Atlantic Ocean to the Pacific Ocean, to speed up shipping of goods between the east and west coasts of the United States and to Asian ports [1].

A canal across the Isthmus of Panama was an ideal solution but the jungle and climate of the Isthmus was also an ideal environment for mosquitoes. Malaria and yellow fever existed there in abundance. French Canal construction efforts began in 1881 but disease decimated the workforce. The French effort eventually collapsed and ceased in 1889 due to financial mismanagement and also because the reputation of disease precluded attempts to draw in new workers [2]. Whilst malaria was hospitalising thousands and caused a greater toll in lives than any other one disease, its lower mortality rate did not strike such fear into the populace as did yellow fever. In 1904, directed by United States Army Surgeon Major W.C. Gorgas, the well-documented construction of the Panama Canal continued. The canal was to eventually extend diagonally across the Isthmus from south-east to north-west, 42 miles from shore to shore. A year earlier, in 1903, Panama granted the United States a concession in perpetuity for a Canal Zone 10 miles wide, 5 miles on either side of the projected Canal line. Whilst Gorgas already had had some experience with the U.S. Army in Cuba in dealing with malaria-carrying *Anopheles* mosquitoes, his main involvement in Cuba had been the introduction of the necessary measures to eliminate the mosquito and thereby successfully handle the yellow fever outbreaks as well as malaria. The military connection in the canal construction was to be maintained with U.S. Army personnel providing engineering expertise.

In 1904, Gorgas set out to test the effectiveness of Major Ronald Ross’s mosquito theory in the Canal Zone that malaria was transmitted by anopheline mosquitoes in a large-scale elimination campaign with thousands of men in ‘mosquito brigades’ working year-long to tackle the mosquito population. Other than a full package of malaria management tools including window screening on the barracks, and mandatory prophylactic use of quinine, his method included reduction in malarial mosquitoes through larval source management. Gorgas did this by clearing wide areas of vegetation, draining swamps, ditching, oiling, and larviciding standing water all along or near the proposed route of the canal. When drainage was not possible along the grassy edges of ponds and swamps, oil was added to the water surface, killing mosquito larvae by blocking oxygen intake through their breathing tubes. When oiling was not suffcient, larviciding was attempted. These efforts greatly reduced malaria incidence, and also greatly increased American chances of canal-building success. Yet malaria continued to challenge, albeit to a much lesser degree during the entire construction programme.

Because malaria management was still in its infancy, effective management methods still had to be developed. The work was carried out in a somewhat haphazard manner. A comment in a 1925 report of the Malaria Commission of the League of Nations hinted at probably why the work was conducted this way. The report referred to Ross as one of the greatest malariologists and yet attributed this statement to him:

*"Amateurs are fond of advising that all practical measures should be postponed pending the carrying-out of detailed researches upon the habits of the anophelines ... and so on. In my opinion this is a fundamental mistake. It implies the sacrifice of life and health on a large scale, while researches which may have little real value and which may be continued indefinitely are being attempted. As a matter of fact, the campaigns at ..., Panama, ... were all commenced before the local carriers were definitely incriminated and their habits studied."* [3]

Ross’ reputation was such that it was likely it provided sufficient weight for his suggestions to have been followed. Accordingly, no baseline anti-malaria survey was carried out before the work began. This would have also explained subsequent comments in a 1925 Proceedings of an Antimalarial Advisory Commission Meeting by Dr Samuel Darling, a pathologist specialising in medical zoology and entomology, and colleague of Gorgas during the canal construction. Darling reportedly mentioned:

*“He [Darling] had an unbounded admiration for engineers but … the engineers [engaged in the anti-malaria work] could not [on their own] fulfil all [the roles necessary for] the work. When they had attempted to do so, much unnecessary work had often been carried out: often water had been drained not calling for drainage, and places filled in not requiring to be filled.”* [4]

Apparently, no entomologist was on hand in Panama to direct or guide the indiscriminate work conducted by well-meaning but entomologically uninformed engineers. However, malaria was not eliminated. The disease was simply controlled to the extent that the construction work could be completed.

Between 1904 and 1914, the Panama Canal was completed. However sustainable malaria control was never considered. No malaria management at the time was conducted outside the Canal Zone and to this day, guidance for travellers to Panama points out that a malaria risk remains, although at least the Panama Canal Zone and certain cities are relatively risk-free.

By way of contrast to the haphazard anti-malarial approach at the Panama Canal, the following malaria control which took place in Palestine was instead dealt with by targeted drainage and also targeted larval source management but all under the direction of an entomologist. By 1918, in the final year of WWI, the British Army under the command of General Allenby had fought its way northwards from Egypt against the Turkish army and had occupied the southern half of Palestine. Palestine was then a severely malarious country and in order to protect the Army from the disease, the British Army under the direction of an entomologist, Major Austen, spent almost 6 months up to 19^th^ September 1918 successfully controlling malaria in the area it occupied whilst it prepared for the final advance against the Turkish Army. After 19^th^ September, when Allenby began his victorious cavalry charge, the anti-malaria work by Austen ceased and the anopheline mosquitoes returned and were again abundant in the previously ‘healthy controlled’ areas. However, because so many thousands of men had been used during the 6 months to control the disease, there was never any suggestion or thought at any time by the British to subsequently sustain or maintain the malaria control activities.

After the 1918 defeat of the Turkish Army by the British Army, the Palestine Mandate, on behalf of the League of Nations, was assigned to the United Kingdom, and operated from 1920 to 1948 by a British civil administration.

## Why Sustainable Malaria Control Became Necessary for the Zionists

Immediately after WWI, it appeared that the question of sustaining malaria control after it had served its purpose was never considered. Malaria merely remained a fact of life.

### The severity of malaria

Modern Political Zionism, the movement for Jewish self-determination in Palestine, arose in the late 19^th^ century as a reaction to anti-Semitic and exclusionary nationalist movements in Europe. Anti-Jewish pogroms in Russia (1881–1884) stimulated growth of Zionism, resulting in the formation of pioneering organisations and the first major wave of Jewish immigration to Palestine. Between 1882 and 1914, approximately 75,000 eastern European Jewish idealists arrived to settle in Palestine, but by 1914, about half this number had died or had left, unable to cope with the severe malarial conditions. To demonstrate the severity of the disease in Palestine at the time of WWI, the following may impress the reader. Allenby was later to comment after his victorious charge on 19^th^ September that he had previously been informed the incubation period for malaria was 7–10 days after being bitten by an infected mosquito. He commented that he had taken this incubation period into account when planning his attack. In fact, the British Army was to decisively defeat the Turkish Army within the 10-day incubation period timeframe from the 19^th^ September onwards when Allenby’s army first crossed the front line, from the ‘healthy’ British Army area into the Turkish untreated positions. Allenby’s calculations were proven to be correct because, approximately 10 days after the initial advance, from 1^st^ October onwards, over 20,000 British troops, over half of Allenby’s army began to collapse or ultimately die from malaria due to exposure to the bite of an infected mosquito beyond the British Army’s ‘healthy’ line. But during those first 10 days, the British Army had a physical advantage by fighting the Turkish Army weakened by continuous exposure to malaria for several months in the untreated Turkish positions, thereby assisting Allenby’s decisive victory.

In 1919, Dr. Manson-Bahr, a former officer in Allenby’s army and a future director of the London School of Hygiene and Tropical Medicine, had described Palestine as one of the most highly malarious countries in the world [5].

### The effect of malaria on the landscape and population of Palestine

The severity of the disease had desolated the whole country, causing many areas to be either almost empty of inhabitants or simply just uninhabitable. Large districts had even been described as ‘useless’ on account of the disease by the Mandate Health Department [6].

In fact, the disease had already decimated the Palestine population to the point that Mark Twain in 1867 wrote of his visit:

*“A desolation is here that not even imagination can grace with the pomp of life and action…We never saw a human being on the whole route.”* [7]

In its 1876 Handbook for Palestine and Syria, the travel agent Thomas Cook & Son said of Palestine that:

*“Above all other countries in the world, it is now a land of ruins. In Judea it is hardly an exaggeration to say that…for miles and miles there is no appearance of present life or habitation, except the occasional goatherd on the hillside, or gathering of women at the wells, there is hardly a hill-top of the many within sight which is not covered with the vestiges of some fortress or city of former ages.”* [8]

The 1922 Palestine Government Census revealed a total of only 389,534 rural/village (mainly Arab) inhabitants for the whole of the country. These rural inhabitants, in fact, represented the majority of a total Palestine population of only 757,182 (103,331 in Bedouin/tribal areas and 264,317 in municipal/ town areas - Jews were mainly part of the municipal/town statistics.)

In 1920, Dr. Israel Kligler, a brilliant Public Health Scientist, a Jewish Zionist, had arrived to settle in Palestine, and was to become the architect of the subsequent malaria control campaign. Kligler wrote of the difficulty of understanding at first sight how a country as sparsely settled as Palestine could have such a disease so widely spread and epidemics so continuous. He wrote that epidemics are usually correlated with crowding. He continued that the answer was furnished by a study of the socio-economic conditions prevailing there, and that because the country was small and undeveloped, there was a constant active movement of the various population groups including the Bedouin and those on annual pilgrimages. He noted that this movement was as effective in spreading malaria and maintaining its epidemicity as it would be in the case of any other infectious disease [9].

**Figure 1:**
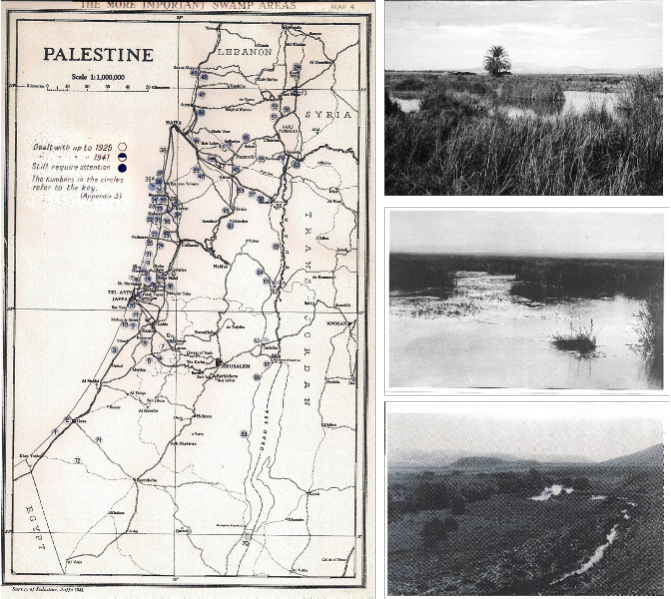
The Palestine Department of Health map of Palestine in 1920 showing the 74 more important swamp areas (numbered) in the early 1920s (with examples of the swamps).

The Mandate Health Department in the 1920s prepared a map of the more important swamp areas in Palestine and this map together with photo examples of the swamps may assist the reader in appreciating the desolation that existed in the country at that time due to malaria (Figure 1).

Therefore Jewish Zionists seeking to settle in Palestine realised they had to somehow undertake steps to control malaria or they would perish. Malaria control had become a priority for them.

## How Sustainable Malaria Control was Achieved by the Zionists

During WWI, up to 19th September 1918, Allenby’s Palestine front had been the only malarious front to successfully control the disease.

Immediately at the conclusion of WWI, sustainable rural malaria management by a civil authority in Palestine had been viewed by the British governing authorities as financially impossible, because it would have required considerable manpower for many years. Indeed, there doesn’t appear to be any record at that time of consideration given anywhere to malaria being sustainably controlled. Apparently, such matters were not then on anyone’s agenda.

In 1922, under Kligler’s direction, the first start anywhere began in Palestine of what was to become a successful national sustainable malaria control campaign, with Kligler’s method of control being basically very similar to that of Allenby’s. However, Allenby could call on an army of thousands to deal with the necessary steps required for malaria control, and which army obviously included personnel obliged to act on Allenby’s directives and orders. Kligler on the other hand was without such an army. If Kligler had later theoretically wished to conduct an identical method to that of Allenby, Kligler would have been obliged to take steps to create a substitute army by trying to engage with (and enthuse) all inhabitants. Further, because initially the number of Palestine inhabitants was then extremely small, Kligler would probably have even had to introduce additional labour from outside the country, as had Allenby in addition to his army when he brought in thousands of the Egyptian Labour Corps.

Immediately after WWI, the problem of rural malaria in Palestine was acute, but it was then never a priority for the Palestine Health Department. In any event, the Health Department did not have the means for conducting an intensive study of and campaign against malaria in the rural community as a whole. However, the control of malaria was an obvious priority for the Zionists because unless the disease could be controlled, the possibility of a Jewish Homeland would have been unlikely.

The American Jewish Joint Distribution Committee (JDC), the leading global Jewish humanitarian organisation, noted the situation and accordingly stepped in to assume financial responsibility for the furtherance of rural sustainable malaria control in the country.

With a view to assisting the Jewish population as well as relieving the government of the embarrassing position, the JDC placed at the disposal of the Health Department a complete organisation for the study of malaria in Palestine and methods of control. Sufficient funds were provided to carry on this task for a period of a little over four years [10]. Because Kligler viewed the malaria work as a public health issue, Kligler agreed these funds should be channelled through the Mandate Health Department.

In 1922, the Malaria Research Unit (MRU) was established by Kligler and funded by the JDC for the special purpose of studying ways and means of controlling malaria in Palestine, for putting the results of these investigations into practice, particularly in the rural areas affecting Jewish settlements either present or proposed and also affecting Arab communities (if any) in the neighbourhood.

A significant moment (at least in our opinion) relevant to this paper had occurred during October 1918 as Allenby’s troops were vacating areas where they had been working to destroy the mosquito breeding sites during the previous six months. A 1919 British Army Report of Allenby’s malaria control [11] noted the Arab mayor of Ramallah, a village north of Jerusalem, had stated that in earlier years, nearly every inhabitant usually went down with malaria during previous summers but the inhabitants of the village had never enjoyed such good health as they had done that year, in 1918. The mayor continued that the inhabitants were so appreciative of what had been done, they were prepared to pay the young soldier who had dealt with the anti-malaria work to return each summer and repeat whatever he had been doing during the recent previous 6 months. There is no record of the soldier having taken up the offer, but it did demonstrate that if inhabitants were exposed to the benefits of controlling the disease, they could be more interested in malaria control rather than resignedly accepting the disease as a fact of life and assuming nothing could be done.

But it also demonstrated a lack of understanding on the part of the mayor and the inhabitants to consider such a task was within the capabilities of the soldier alone. As if to highlight the general ignorance in dealing with malaria, Austen, Allenby’s entomologist, included the following comment in a lecture [5] which he gave in 1919 about his malaria experience in Palestine:

*“It is perhaps worth mentioning that an inhabitant of RamAllah, a Syrian medical student, on being shown living larvae of Anopheles bifurcatus, said that they had nothing to do with malaria mosquitoes, and were to be found in every cistern in the village!”* (The exclamation mark was added by Austen)

Austen seemed to make the point it was a medical student, not just an uneducated inhabitant, who had made the remark as if to emphasise the lack of knowledge about malaria existed at all levels.

Allenby had only needed malaria control for a few months before his victorious advance on 19^th^ September. Kligler on the other hand needed permanent sustainable malaria control to make Palestine habitable, no longer desolate. Kligler required the inhabitants for years to come to co-operate in the maintenance of the anti-malaria works which would have included ensuring those mosquito breeding sites that were to be destroyed by Kligler in fact would remain destroyed. Kligler’s aim was to turn a country that had hitherto been almost empty for centuries into a place where people could live safely, without disease.

Kligler would have seen and read the British Army malaria report and also Austen’s lecture paper and would accordingly have noted the lack of knowledge concerning malaria on the part of the inhabitants. This is an important point because, as will be shown, Kligler’s major contribution to sustainable malaria control was his emphasis on education for all inhabitants about all aspects of the disease.

Over the next few years, Kligler wrote outlining his basic strategy he intended to employ for controlling malaria and its sustainability in Palestine. He would usually refer to his subsequent experimental demonstrations which included details of the anti-malaria campaign and also the education. Such were the matters considered in his demonstrations:

*“The greatest emphasis was laid on measures against mosquito larvae. … The procedure consisted in a thorough survey of the area, the emptying of all water-containing receptacles, the closing of cisterns or water barrels where possible, fixing leaking pipelines and petrolising wadis, pools, etc. Slowly, as cooperation could be obtained, some wadis were cleaned and some of the smaller swamps dried. But the chief reliance was placed on regular and thorough inspection and on petrolization.”* [12]

*“An important element in these demonstrations was the educational propaganda carried on along with the work. … For the first time, malaria assumed the importance of a real and preventable disease which should be eradicated. … The remedies for the malaria situation in Palestine are briefly; (1) the cooperation of the various settlements in the cleaning of their long-neglected and consequently overgrown wadis. Once properly cleaned and regulated, these wadis could be kept under control at very little expense; (2) the improvement and control of the method of irrigation. … ; (3) the gradual drainage of the swamps resulting from underground seepage, overflowing springs, etc., by the inhabitants in cooperation with other agencies. …(4) the constant supervision of all these places to protect the inhabitants against their own negligence and indifference and inculcate the habit of proper self-care. Education is as important in malaria control as in other phases of Public Health Work.”* [12]

Much was written by Kligler that the educational aspect of the work was certainly as important, if not more so, as any other. Theoretically, inhabitants should have wished to be free of the disease and come to witness the demonstrations which were ultimately for their benefit. But in practice, however, Kligler instead still had to work to induce the inhabitants to attend the demonstrations. So how was that done?

The 1923 Annual Report of the MRU for the Palestine Department of Health [13] explained how the inhabitants were moved to attend the demonstrations:

*“First in importance among the activities of the Unit were the Malaria Control Demonstrations, conducted in various parts of Palestine, in order to …. establish definitely the possibility of large-scale malaria control in Palestine at a low cost. … [These demonstrations] also served as an excellent means of practical education of the public at large in the value of malaria control. The educational value of the demonstrations is perhaps equal in importance to the immediately practical results obtained. …*

*Another phase of the activity of the [Unit] was the permanent elimination of breeding places. … we attempted to stimulate co-operative drainage undertakings by the villages and settlements. We offered to contribute part of the cost of the work on condition that the inhabitants of the communities affected contribute the rest either in money or in labour. This procedure helped to awaken the interest of the inhabitants in their malaria problem and has already borne fruit in several completed and projected drainage schemes. This year’s experience demonstrated that many of the important swamps now existing in the Demonstration Areas can be eliminated at a relatively small cost, and that small grants given to those settlements ready to bear a part of the cost will help them to remove permanently at least some of the chief sources of malaria.”*

Presumably, if the inhabitants saw the authorities were prepared to make a serious commitment by paying towards the inhabitants’ safety and wellbeing, freeing them from malaria, malaria would have suddenly assumed the importance of a real and preventable disease which could be effectively controlled. It is likely that any attempt to merely buy the inhabitants’ long-term cooperation without the inhabitants also being made to feel involved would have been ineffective. Because the inhabitants were also contributing, they were therefore involved and therefore would also have had an interest in the project’s success.

In 1925, Dr. Paul F. Russell, director of the Rockefeller International Health Division after he had inspected the anti-malaria work in Palestine, wrote:

*"I do not know when I have seen better and more successful anti-malaria work than that which is being done in Palestine (…). The co-operation of the people with the authorities leaves nothing to be desired (…). It is an ideal way to carry out malaria work because it makes the population served by the anti-malaria measures participants in the project from the beginning; they then have a better understanding of the problem and will be more ready than they otherwise would be to take care of the necessary maintenance."* [14]

If ever proof was needed of a population’s commitment of wholeheartedly supporting the malaria control program, then the subsequent co-operation that existed in Palestine is that proof. In 1941, the British Mandate’s Department of Health had reviewed the malaria position in Palestine, and praised the co-operation:

*“In the true rural areas the lasting effects of swamp and marsh reclamation, the proper irrigation of gardens and similar areas, and the periodical canalisation, filling and clearing of stream beds, have so impressed the people by the resulting improvement in health, and the land thus made available to farmer and shepherd, that their prompt and energetic co-operation is one of the most remarkable features of the antimalarial campaign in this country.”* [15]

## An Interest in Malaria Control was (and Still is) Vital

Whilst Allenby/Austen could order and insist work be conducted in a particular manner, Kligler instead aimed/hoped for inhabitants to cooperate because they had understood why it was necessary to deal with a matter in a particular way. He treated all inhabitants (Jews and Arabs) with dignity and respect, he educated them and returned again and again, if necessary, to repeat such explanations if something was not at first understood, and thereby he obtained their willingness and interest to conduct the work in a manner he required.

It cannot be stressed too strongly that both Allenby/Austen and Kligler had recognised and relied upon all steps in the control of the disease to be conducted thoroughly. But for sustainable malaria control (*and it is just as important for the malaria community today to appreciate and understand*) Kligler’s success was greatly due also to the inhabitants willingness to **continuously** conduct all tasks thoroughly over prolonged periods, otherwise the mosquitoes and the disease would return. Such thorough work was obviously expected by Kligler because the inhabitants would have already seen and knew the standard of work required due to Kligler’s demonstrations.

To ensure all tasks were carried out correctly and thoroughly, Kligler wrote the actual success of any control measures also depends on the quality, training, and dependability of the local inspector. The selection and training of this class of personnel was, therefore, of the utmost importance in malaria control. The man should have been sufficiently intelligent to grasp the elements of the theoretical basis of the work and become interested in the works as such, and to understand the responsibility resting on him. He should also have been ready to do the work, and not merely stand by and order someone else to do it. Kligler wrote these were considered essential qualities, and only such men were selected who possessed both of them [16].

## Encouraging Participation

**Figure 2:**
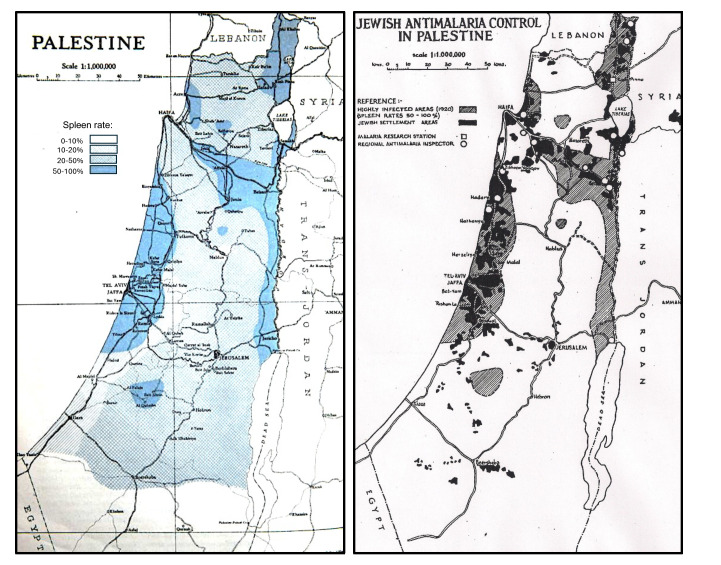
Spleen rates from 1920 (left image), indicating the severity of malaria, clearly overlap with lands purchased by Jews (right image, black areas).

Because the Jewish settlements were in some of the most severely malarious areas, all Jewish inhabitants were more than likely willing to be involved in the malaria control procedures. In 1920, a map of malaria severity in Palestine was drawn (Figure 2, left) by the British Mandate Department of Health [15]. This map designated the worst areas (dark blue; spleen rates ranging from 50-100%), highly malarious, and the Department of Health even declared some of these areas as uninhabitable. The superimposed UN 1947 map (Figure 2, right) demonstrates the extent to which lands purchased by the Jews (in black) were in these previously malarious areas, usually the only lands available to them.

Whilst the JDC funding for the work was initially intended to assist the struggling Jewish settlements’ control of the disease, Kligler explained in 1925:

*“Insofar as the need required and the means permitted the control was extended to the neighbouring Arab settlements [that may have then existed] with a view slowly to extend the work to cover the entire country.”* [17]

The establishment of the MRU relieved the Palestine Mandate Government Health Department of a part of the task which should rightly have been performed by it. The necessity for all the rural anti-malaria work to have been financed mainly by Zionist charitable funds for the first years was an obvious indication in the first place of a lack of priority on the Mandate’s part in dealing with rural malaria.

It had been understood from the beginning in 1922 that as soon as the pioneer work was done and conditions improved, the Health Department would assume an increasingly larger share in support of the necessary supervision and control. After four years, the Mandate Government accordingly entered into negotiations with the JDC to gradually share the expense for the next several years, and then for the Government to eventually assume full responsibility. Kligler wrote in 1930:

*“All this [malaria control] was achieved almost entirely by educational methods and moral suasion. … But even more telling is the fact that all the [Jewish settlements] and some of the [neighbouring] Arab villages soon learned to appreciate the value of the work and spent collectively thousands of dollars annually for control work. … By 1926 the main features of the problem had been revealed and effective methods of control evolved. The responsibility for the practical control work was thereupon taken over by the Government Department of Health where it belonged, the JDC continuing to cooperate by annually decreasing grants which supplanted the Government expenditure.”* [18]

However, to illustrate how significant and important the level and standard of thoroughness remained for Kligler, the following may assist.

Kligler was worried the Government would consider itself entitled to begin choosing the inspectors when it eventually was to begin contributing to the anti-malaria funding. Kligler was at pains to point out that the practical maintenance, supervision and control of the rural anti-malaria work was essential. Until that moment, during the initial four-year funding period, Kligler would have had effective control of the quality and standards required of the malaria inspectors. But if the expense was to be subsequently shared between the JDC and the Government, Kligler could have expected some of his control over the quality of inspector to be diluted because of the Government’s tendency to cut back and economise thereby forfeiting high standards. Already, Kligler had previously tried out one of the Government Health Department’s best inspectors since the Department had begun to recruit for general tasks. But, according to Kligler, the ‘best inspector’ provided by the Health Department had ‘failed miserably’. Kligler had commented that the Health Department inspectors, as compared to Kligler’s own group of inspectors, were cheaper because they were of a lower grade of intelligence, had a poorer education and training, with no real understanding of the work, and no interest in it.

Such a lack of interest could be explained as follows. Malaria control was a priority, a necessity, for the Zionists but was not a priority for the British. Kligler’s inspectors were all as committed as Kligler to the success of malaria control whereas the Mandate’s Health Department’s workforce was not obliged to have that same commitment and therefore usually lacked the interest in the project that Kligler required.

Kligler therefore had to think up an arrangement for the JDC to partially contribute towards the malaria control work, yet ensure the necessary work was conducted thoroughly and up to his own required standard. If the Government was to be paying for the work out of its own pocket, Kligler had felt it likely the Government Health Department, in its attempt to reduce expenditure, would introduce cheaper, poor-quality inspectors into the control, thereby enabling sub-standard work to be accepted. As a solution however, Kligler managed to obtain agreement that in respect to areas which were controlled by his MRU and were eventually found to be sufficiently developed, only then would those areas be handed over for control by the Government Health Department sub-inspectors [10].

## International Recognition

In 1925, Kligler’s methods were internationally recognised. That year, the League of Nations Malaria Commission had been informed of anti-malarial works then taking place in Palestine and the Commission came to inspect. The Commission’s subsequent Report [3] concluded with:

*“(…) the work done in Palestine, by destroying pessimism, raising hopes, (…) becomes a welcome and invaluable addition to practical malariology, and the men who carried it out can be regarded as benefactors not only to the Palestine population but to the world as a whole.”*

The President of the Malaria Commission, Professor Nocht, was so impressed, he separately commented at the end of the Commission’s stay/inspection in Palestine:

*“Palestine showed the fruits of an energetic and victorious campaign which would stimulate others to follow the methods there employed.” And “It was not the custom of the Commission to make comparisons but he would on this occasion, say that the interest that Palestine had provided was unsurpassed by that of any of their other visits [to other countries]. (…) the Commission would (…) greatly profit by its visit to Palestine, and the world would surely benefit by what they had seen there, through the medium of the League of Nations.”* [4]

## The Relevance of Education

As already mentioned, Kligler had pointed out that the educational aspect of the malaria control work was certainly as important, if not more so, as any other. He accordingly set about engaging with the inhabitants. And that engagement was strong and effective. But was the importance of this step understood or appreciated 100 years ago? Is it even understood or appreciated today?

The fact that Kligler managed at all to overcome immense difficulties in educating the inhabitants was a triumph, but the education was hardly noticed outside of Palestine in 1922. Even if other countries had been aware of the education, they would have probably dismissed the education as having no link or connection with malaria control and that it was even pointless or irrelevant.

Kligler pressed on with the difficult task of ensuring each inhabitant understood and appreciated the steps required in the anti-malaria work. There was no such thing as a Palestinian identity or people, and because there were different cultures and backgrounds, that meant there was no standard approach for the instructors to follow. Kligler wrote in 1928:

*“Palestine is a country of mixed peoples, many religions, and all gradations of civilization. There are Jews from the Orient and the Occident, and side by side there are city Arabs, the peasants, and the roving Bedouins. There are Moslems, Jews, and Christians, people of every sect and denomination. And there are as well all gradations of culture, from the Nomadic, pastoral Bedouins to the most modern industrialised groups. A more heterogeneous population can hardly be found anywhere in the world.”*[9]

Before WWI and as the Ottoman Empire contracted, several Muslim communities were expelled from those other parts breaking away from the Ottoman Empire. These communities included Circassians (i.e. probably 1.5–2 million Muslim refugees from the Caucasus, Russia), Algerians, and Bosnians, and were periodically ‘resettled’ by the Ottoman Empire into the region, with some of these refugees being sent to Palestine. There were desolate areas in Palestine, and it didn’t seem to trouble the Ottoman authorities where these Muslim exiles were placed. Kligler even commented in 1928 of his early experience in Palestine concerning these Moslem exiles:

*“One passes a Circassian village half encircled by marshes – one of the outposts colonized by the Turks forty years ago with hardy stock from Circassia, nine hundred strong. Now there are scarcely fifty souls left in the village, mostly native stock and all stamped with the effects of malaria. This condition is repeated at Ramadan, Jelile, at Nebi Rubin, in short, all along the coast.”*[9]

With different cultures and backgrounds, with each inhabitant receiving such attention to ensure he understood the work ahead, a great deal of energy and time was expended by Kligler with education. Sustainable malaria control was a priority for Kligler and he recognised such education was necessary to ensure the inhabitants’ full co-operation.

Therefore, the reader should recognise how arduous, time consuming and difficult it must have been for Kligler to organise the education, but it was a necessity, it had to be thorough and as a result, it was very effective. It is very important for the reader to appreciate the education was not some casual peripheral undertaking.

Kligler sadly died from a heart attack in 1944 and therefore never lived to see the elimination of malaria in those areas where he had worked. He had always recognised and emphasised that it was vital to continually ensure all inhabitants maintained an interest in the control, even when the disease seemed to have disappeared. Kligler taught the disease would return if the destroyed mosquito breeding sites were not maintained, so the inhabitants had to ensure the destroyed breeding sites remained destroyed. To have the anti-malaria work continuously maintained. Kligler emphasised the necessary work required:

*“… continuous supervision. {He} pointed out the work of the inspector, as well as that of the physician, had to be checked at frequent intervals, particularly during the early stages. This also had an educational value because it kept the question alive, …”.* [9]

It was fortunate that by the time of Kligler’s death, most inhabitants had been treating any anti-malaria steps as a habit, and therefore any anti-malaria directives by the Mandate Health Department would likely have received the inhabitants’ usual cooperation thereafter.

So why hadn’t Kligler’s method been imitated elsewhere?

Kligler’s sustained malaria control was bound up with cooperation or engagement with the community. Palestine was desolate when Kligler began his work, the country was then almost empty of inhabitants in many areas, and therefore other malarious countries may perhaps have wondered how successful his initial approach of community engagement would have been had there been a significant population when he began.

Also, Kligler’s approach must have appeared very radical for the time. There was no problem for the general malaria community in accepting his basic method of control of the disease, but his view on how to continuously maintain that control was to become controversial or appear unrealistic. In 1927, the League of Nations issued a Report to the Health Committee by Dr. A. Lutrario on the Work of the Malaria Commission:

*"... the sole purpose of the campaign against malaria, as far as the Commission is concerned, is to reduce the incidence and severity of the disease. Measures designed to accomplish more than that (particularly measures aiming at eradication) are not a wise proposition ... The Commission had occasion to enter into relations with a number of states which ... had undertaken an energetic campaign against malaria. The disease yielded to the measures which were taken, but reappeared with added virulence as soon as these measures were somewhat relaxed. This is a very serious result which brings in its train disillusion and discouragement."* [19]

Kligler’s antimalarial approach was new and untested. Whilst not mentioning Kligler or Palestine by name, Lutrario appeared pessimistic about those few Palestine inhabitants continuing to maintain the works of ensuring the breeding sites remained nonproductive or destroyed. Obviously Lutrario had no idea or grasp of how effective Kligler’s education was.

If it hadn’t already existed in some minds, Lutrario’s statement may well have created doubts as to the likelihood of success for Kligler’s approach. With doubts then suggested by the experts at the League of Nations about the success of Kligler’s sustainability, others would have wondered how different methods could achieve such sustainability. There is no direct evidence that Lutrario’s statement was a significant turning point, but the shift from praise in 1925 by the League of Nations Malaria Commission to Lutrario’s pessimism does indicate this may well have been the case.

Also, Kliger’s gentler and gradual approach of community participation may have appeared out of step with the ‘rougher, tougher’ social and political currents of the day, and it is well to remember the changes taking place at that time within Europe also included the harsh violent rise of Mussolini and Hitler.

As an example of the changes in method, an alternative vector control strategy to that of Kligler’s was successfully applied by Dr. Fred L. Soper, an eminent American epidemiologist and public health administrator, in the 1930s and ‘40s in Brazil and Egypt, respectively. Community engagement did not feature prominently on his agenda. His view on how to achieve the desired result was militaristic. He believed in success only based upon meticulous planning and rigorous execution in the destruction of the mosquito breeding sites. He would work with local inhabitants but only upon decent training, and they would be paid as employees, and not work on a voluntary basis. He believed in vertical programmes, top-down not bottom up. Although both Kligler and Soper fought malaria, a critical difference in approach lies in the fact that Soper focused on eliminating the vector (and thus the disease) whereas Kligler focused on eliminating the disease (through controlling the vector). A subtle yet important point to consider, since vector elimination (Soper) requires an areawide approach, which will be much harder to accomplish when working with community volunteers (Kligler). It is an extremely valid and realistic point that can be made of when a disease has almost (but not completely) disappeared people at all levels (from the community to the highest political echelons) lose interest and start to focus their energy on other issues. Therefore, Soper’s view had to be taken very seriously, namely that of reaching ‘zero’, of achieving malaria elimination, would be next to impossible when based on community participation.

Soper’s view today would presumably have included paying young people from around that area to be trained to do the job and they should continue doing it until ‘zero’ was reached. In today’s world, ‘zero’ would probably not be viewed as vector elimination but disease elimination resulting in ‘anophelism without malaria’. As such, hybrid models, which incorporate Kligler’s community-based approach with the rigour of Soper’s style to walk the last mile, deserve to be investigated today.

Notwithstanding Lutrario’s pessimistic forecast, Kligler was not distracted and maintained his approach and method without change but it wasn’t until 1967 that the World Health Organization (WHO) declared malaria as eliminated in Israel.

### Rate of Arab natural increase

Obviously Kligler’s method worked, it was proven successful because Israel was declared free of malaria in 1967 by the WHO. But the effectiveness of Kligler’s education was also demonstrated in an unanticipated and surprising way.

In 1925, the League of Nations Malaria Commission had heard of the successful anti-malaria works underway in Palestine, and came to inspect. A comment in the subsequent 1925 Malaria Commission Report after the Commission had inspected the anti-malaria works was of very great signifcance:

*“Above all, it has succeeded in inducing the people of the country to take an interest in health problems and to co-operate in measures for the prevention of disease.”* [3]

Without Kligler’s education, the 1925 Report wouldn’t have been able to make such an observation. In 1938, a British Government Commission reported:

*“(…) an abnormally high (and possibly unprecedented) rate of natural increase in the existing indigenous population.”* [20]

This fact had not in previous years generated great interest, but now the Commission Report continued by referring to the:

*“(…) astonishing change in the Arab population since [WW1] (…)”*

and further added:

*“It would seem that the population growth must be due mainly to a lower death rate, brought about (…) by general administrative measures, such as antimalarial control (…).”*

The link of the extraordinary population increase to malaria elimination had been ever present, but was rarely mentioned. A previous 1937 Palestine Royal Commission Report [21] had included:

*“The growth in [Arab] numbers has been largely due to the health services, combating malaria, reducing the infant death rate, (…).”*

This rate of increase in the population was not just a casual or passing local impression and was to be noted elsewhere. Thus Palestine, a previously almost-empty desolate country, was to experience an ‘abnormally high rate of natural increase’ and ‘astonishing change in the population’ which was confirmed in the Statistical Year Book of the League of Nations 1931/32 as the highest in the world [22].

The 1938 Commission was obviously quite surprised by the population increase and also by its repeated use of the description ‘abnormal’. It mentions:

*“As the result of the abnormally high birth rate and the relatively low death rate, the natural increase of the Arab population is abnormally high.”*

Thanks to sustained malaria control, Palestine after WWI generally became a new experience for both Arabs (due to greatly increased numbers) and also Jews (due to availability of habitable land). Neither Jew nor Arab could have been prepared for the new circumstances. The numbers of ‘new’ Jews and ‘new’ Arabs greatly exceeded the pitiful numbers of Jews and Arabs that had managed to barely exist in Palestine in 1918. The pre-WWI Palestine bore hardly any similarity or resemblance to the post-WWI Palestine, not only in relation to the numbers of Jews and Arabs but also in relation to the extent of reclaimed and available usable and habitable land. Whilst the increase in Arab numbers would also have included Arabs entering Palestine, even possibly illegally, the bulk of the increase in Arab numbers was made up by natural increase. Based on the United Nations Jews and Arab/non-Jews figures, interestingly, the great increase in numbers of both these ‘new’ Jews (524,536) and ‘new’ Arabs (563,842) during the period 1918-1947 was almost the same for both communities [23].

Almost all historical narratives fail to explain why, from 1918, from the end of WWI onwards, there were later so many more Arabs in 1948 at the time of the creation of the State of Israel than there were in 1918. Very few appear to notice, and even fewer ask why.

## Looking Ahead

For those concerned with community engagement or participation, the following may be of interest.

It is perhaps helpful for the reader to visually imagine components that may be relevant in malaria control and elimination. Also, the following sets out a suggested weight/importance that is often associated with each of these components. The components were explained in two Ifakara Health Institute Master Classes in 2021 [24,25] on the topic of mosquito bednets with the comment that 20% of solutions to malaria elimination involved technology, knowledge and science, and that the remaining 80% of solutions involved ‘Improved management and better engagement with the Community’. Regarding this 20:80 distribution, it was commented [25] that 80% was the correct weight. The magic bullet on its own is harmless, but becomes highly efficacious when used with the 80% magic gun of a health and social system to bring it to, and ensuring that it is accepted by, the people.

It is little appreciated today that one hundred years ago, Kligler had already recognised the weight and significance of the 80% component. How one taps into that 80% part would depend upon how the malariologist viewed and read that particular community. When Kligler first began, he read those few Palestine inhabitants well, and malaria elimination was the outcome.

There was, and still is, no ‘quick-fix’ for the elimination of the disease. A major point which seems to be overlooked is that Kligler did not attempt to introduce an instantaneous blanket malaria control over an entire region. His was a gradual approach for those inhabitants who had ‘signed up’ for it, and in that way he could secure the malaria control that had just been achieved for that location.

So has the malaria community learnt anything from Kligler’s methods? Or from Soper’s approach? Perhaps their methods at that time, either community-based or militaristic, were, and maybe still are a little too radical for today’s malarious countries to digest. And perhaps the following can explain. The WHO Handbook on Integrated Vector Management (IVM) included a brief historical note about malaria elimination. It merely stated:

*"Before the Second World War, vector control was conducted predominantly by environmental control of the proliferation of mosquitoes. [e.g., the 1922 Palestine method] .... There is evidence that environmental management had a clear impact on disease; however, elimination of disease was never on the agenda. The advent of DDT and other organochlorine pesticides during the 1940s changed this situation. ... The focus of vector control on insecticides meant that environmental management and other alternative methods were underexploited or even forgotten."* [26]

Although a few countries have freed themselves of malaria in recent years, progress in sub-Saharan Africa has stalled for nearly a decade. The world’s focus on the delivery of commodities (like bednets) was successful up to 2016, but later proved insuffcient to eliminate the disease. Now that even commodity financing is threatened by global budget cuts under the Trump administration, it is high time to change our strategy. From commodity-driven to strategy-driven. A closer look at how pioneers in malaria elimination, like Kligler and Soper, became so successful is therefore more than warranted. An understanding of what formed the basis of their success is not just an exercise of interest to historians but should form the groundwork of moving forward towards a world free of malaria.

## References

[1] Dunkel FV, Alexander A: (2020). Three stepping stones leading to malaria elimination, changing world maps on the way.. MalariaWorld J..

[2] Avery RE: (1913.). The French Failure: America’s Triumph in Panama. IL,. https://tinyurl.com/yck9fchd.

[3] League of Nations: Health Organisation. (1925.). Malaria Commission. Reports on the Tour of Investigation in Palestine in 1925..

[4] Palestine Antimalarial Advisory Commission: (1925,). Proceedings of the 11th Meeting of the Palestine Antimalarial Advisory Commission, 19 May 1925.. Abstract in: Trop. Dis. Bull..

[5] Austen EE: (1919). Anti-mosquito measures in Palestine during the campaigns of 1917–1918.. Trans. R. Soc. Trop. Med. Hyg..

[6] Palestine Department of Health: (1941). A review of the control of malaria in Palestine (1918-1941)..

[7] Twain M: (1869). The Innocents Abroad.. https://tinyurl.com/2x463yx8.

[8] Cook T, Son: Cook’s Tourists’ Handbook for Palestine and Syria.. http://tinyurl.com/37zt2tcv.

[9] Kligler IJ: (1930). The epidemiology and control of malaria in Palestine..

[10] Kligler IJ: Letter dated 31st December 1925 to Flexner, and agreement of 7th January 1926 between Colonel Heron and Dr Kligler..

[11] Angus W: (1919.). Report on malaria in the Egyptian Expeditionary Force during 1918.. Wellcome Library, 1919, ref. WTI/RST/H/3, January.

[12] Kligler IJ: (1924). Malaria control demonstrations in Palestine: 1. Malaria control and its cost.. Am. J. Trop. Med..

[13] Palestine Department of Health (Kligler, I.J). (1923). Malaria Research Unit, Haifa, Annual Report, 1923.Maintained by the American Joint Distribution Committee in co-operation with the Government of Palestine..

[14] Russell PF: (1925). Rockefeller Foundation Archive Centre, RFA,.

[15] Palestine Department of Health: A review of the control of malaria in Palestine (1918-1941). (1941).

[16] Kligler  IJ: (1930). The epidemiology and control of malaria in Palestine.. http://tinyurl.com/mwv8f9ur.

[17] Kligler  IJ: (1925). The fight against malaria.. Menorah J..

[18] Kligler  IJ: (1930). Fighting malaria in Palestine. The Jewish Social Service Quarterly. Jewish Communal Service Association of North America (JCSA), National Conference of Jewish Social Service.. September.

[19] League of Nations: Report to the Health Committee by Dr. A. Lutrario on the work of the Malaria Commission. (1927). League of Nations Health Organisation, Geneva, Nov..

[20] Palestine Partition Commission Report Presented by the Secretary of State for the Colonies to Parliament by Command of His Majesty, October 1938. (1938.).

[21] Palestine Royal Commission Report Presented by the Secretary of State for the Colonies to Parliament by Command of His Majesty, July 1937. (1937).

[22] Statistical year-book of the League of Nations. http://tinyurl.com/5bt8rvcn.

[23] United Nations Conciliation Commission for Palestine: (1946). Settled Population of Palestine by Town and Sub-District, Estimated as at 31st December.

[24] Ifakara Health Institute Two Billion Mosquito Nets & Counting: a MasterClass with Prof. Christian Lengeler.. http://tinyurl.com/39d4ee6x.

[25] Ifakara Health Institute: Malaria Strategies: A MasterClass with Prof. Marcel Tanner. https://tinyurl.com/3evshhz5.

[26] World Health Organization: Handbook for Integrated Vector Management. Geneva, World Health Organization, (2012). http://tinyurl.com/dx4p86x6.

